# Biodegradable Guar-Gum-Based Super-Porous Matrices for Gastroretentive Controlled Drug Release in the Treatment of *Helicobacter pylori*: A Proof of Concept

**DOI:** 10.3390/ijms24032281

**Published:** 2023-01-23

**Authors:** Roberto Grosso, Elena Benito, Ana I. Carbajo-Gordillo, M. Gracia García-Martín, Víctor Perez-Puyana, Pablo Sánchez-Cid, M.-Violante de-Paz

**Affiliations:** 1 Departamento de Química Orgánica y Farmacéutica, Facultad de Farmacia, Universidad de Sevilla, C/Prof. García González, n.º 2, 41012 Sevilla, Spain; 2 Departamento de Ingeniería Química, Facultad de Química, Universidad de Sevilla, 41012 Sevilla, Spain

**Keywords:** semi-IPN, super-porous hydrogels, guar gum, bioorthogonal chemistry, gastroretentive DDS, *Helicobacter pylori*, mucoadhesive polymers, amoxicillin, vonoprazan

## Abstract

An increase in resistance to key antibiotics has made the need for novel treatments for the gastric colonization of *Helicobacter pylori* (*H. pylori*) a matter of the utmost urgency. Recent studies tackling this topic have focused either on the discovery of new compounds to ameliorate therapeutic regimes (such as vonoprazan) or the synthesis of gastroretentive drug delivery systems (GRDDSs) to improve the pharmacokinetics of oral formulations. The use of semi-interpenetrating polymer networks (semi-IPNs) that can act as super-porous hydrogels for this purpose is proposed in the present work, specifically those displaying low ecological footprint, easy synthesis, self-floating properties, high encapsulation efficiency for drugs such as amoxicillin (AMOX), great mucoadhesiveness, and optimal mechanical strength when exposed to stomach-like fluids. To achieve such systems, biodegradable synthetic copolymers containing acid-labile monomers were prepared and interpenetrated with guar gum (GG) in a one-pot polymerization process based on thiol-ene click reactions. The resulting matrices were characterized by SEM, GPC, TGA, NMR, and rheology studies, and the acidic hydrolysis of the acid-sensitive polymers was also studied. Results confirm that some of the obtained matrices are expected to perform optimally as GRDDSs for the sustained release of active pharmaceutical ingredients at the gastrointestinal level, being a priori facilitated by its disaggregation. Therefore, the optimal performance of these systems is assessed by varying the molar ratio of the labile monomer in the matrices.

## 1. Introduction

In 2021, 26,560 new cases of gastric cancer were diagnosed in the United States [1]. This positions stomach neoplasms above larynx, ovary, and esophagus cancers in terms of incidence in the studied population. The most important risk factor reported for gastric cancer is infection by *Helicobacter pylori* (*H. pylori*), which is the pathogen that should be primarily addressed in gastric cancer prevention programs [2]. 

Currently, antibiotic-based therapies against this pathogen are not foolproof, especially considering the dramatic increase in antibiotic-resistant *H. pylori* strains. Thus, for example, some studies have pointed out that it is not possible to eradicate the bacterium in at least 2 out of 10 patients treated [3], leading to soaring resistance to antibiotics, probably due to the fact that combined antibiotic therapies are prescribed systematically when *H. pylori* is detected. In general terms, the ideal anti-*H. pylori* treatment is the combination of two to three antibiotics and a proton pump inhibitor [4]. Out of all the antibiotics analyzed previously, amoxicillin (AMOX) seems to have the lowest prevalence of resistance among *H. pylori* strains (except in Africa), therefore making it an ideal candidate for optimized dual therapy (i.e., amoxicillin and a proton pump inhibitor) to be studied as a first-line treatment for infections in most countries [4]. 

To overcome the drawbacks found in conventional therapies, improvements in anti-*H. pylori* therapies are required. Two main challenges have been identified [4]: (a) the amelioration of active pharmaceutical ingredient (API) combinations, for which the authors have recently proposed the use of vonoprazan fumarate (VONO, also known as vonoprazan) [5] as the optimal drug to be combined with AMOX in *H. pylori* infections [4], and (b) the development of ex-profeso-designed gastroretentive drug delivery systems (GRDDSs), capable of guaranteeing a prolonged and reliable performance of the antibiotic formulation. 

The development of buoyant systems is one of the main approaches to achieving effective GRDDSs [6]. The types of systems that have been designed as AMOX-GRDDSs vary from floating in-situ gelling systems [7,8] to floating beads [9], bilayer floating tablets [10], hollow tablets [11], floating capsular devices obtained by 3D printing [12], floating raft systems [13], and floating microballoons/spheres [14]. To the authors’ knowledge, all the formulations proposed for the manufacture of buoyant AMOX-loaded GRDDSs incorporate at least two or three swelling enhancers of effervescent excipients to guarantee such a feature during the expected release tempo. This current excipient-addition approach is costly and has an ecological impact that should be prevented in future GRDDS designs. It is of fundamental importance to develop systems that are capable of loading large quantities of AMOX, performing at different pHs (1.2 and higher), and are stable and capable of disintegration when the action is exerted. Additional properties, such as mechanical strength to keep them in the stomach when performing their therapeutic role, mucoadhesion, and the capability to encapsulate large drug loads, will be of great significance. The design of matrices with the above-mentioned properties for their use in suitable AMOX dosage forms is the main goal of the present work.

**Figure 1:**
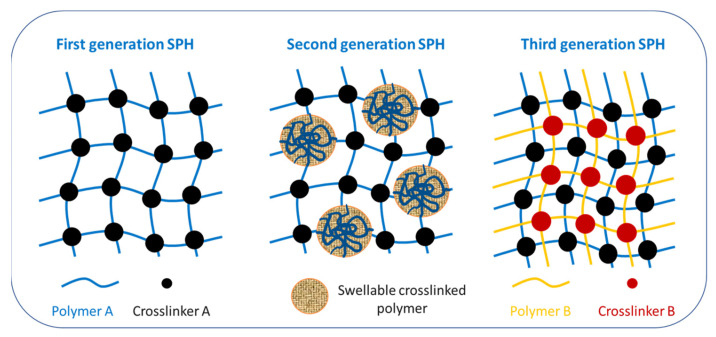
General scheme of the different types of super-porous hydrogels.

Super-porous hydrogels (SPHs) are three-dimensional networks constituted by lightly cross-linked hydrophilic polymers that can absorb, swell, and retain aqueous solutions up to hundreds of times their own weight, within minutes, to reach the equilibrium swollen state regardless of their size in the dried state [15]. They display relevant advantages compared with other hydrogel systems [16], such as the presence of interconnected pores throughout the hydrogel matrices responsible for their superabsorbent properties. The general structure of the different types of SPHs is recorded in Figure 1.

Today, up to three generations of SPHs are available for clinical use, but third-generation SPH hybrids (SPHHs, also called super-porous hydrogel interpenetrating networks or SPH-IPNs) are preferred because they display better mechanical strength [17].

SPH hybrids are prepared by adding a water-soluble or water-dispersible polymer (hybrid agent or Polymer 1) that can be cross-linked through chemical or physical cross-linking after the SPH is formed. Once the second network is formed (Polymer 2), the whole system becomes an interpenetrating polymer network [18]. If one of the constituent polymers is not cross-linked, the system is then known as a semi-IPN.

For the eradication of *H. pylori* infection, the incorporation of mucoadhesive properties into the floating gastroretentive dosage forms is especially advantageous. They can adhere to the stomach wall, survive gastrointestinal motility for a longer period [19], and thus enhance the local action of the drug in the infected area [20]. Guar gum (GG) is a natural polysaccharide that displays interesting mucoadhesive properties; it is degradable under physiological conditions and can be used in GRDDSs [21,22]. 

The general objective of this work is to provide a new set of biocompatible materials for the development of streamlined, sustained-release GRDDSs of AMOX that, when administered with VONO in a combined oral formulation, can effectively eradicate *H. pylori* in the stomach of infected patients. These systems should have a low ecological footprint, be easy to synthesize, and possess mucoadhesive and floating properties by themselves, that is, without the need for any buoyancy-promoting excipients. Additionally, once the GRDDSs have exerted their function, the final material should be biodegradable and easily eliminated from the human body. 

To the authors’ knowledge, there is no precedent for the use of third-generation IPNs for the release of APIs in oral formulations based on thiol-ene click reactions. The use of orthogonal chemistry in the preparation of such materials will provide a wide range of possibilities in IPN-based matrices since innumerable types of natural polymers with unique properties can be combined with a variety of synthetic polymers prepared ex profeso for these systems. The degradability of the materials will be ensured not only by the hydrolysis of the chosen natural polysaccharide but also, and importantly, by the inclusion of labile segments in the second polymer that will form part of the semi-IPN-based matrices. 

To achieve this, several specific objectives have been set:

- Prepare, characterize, and compare different semi-IPNs using GG as the natural polysaccharide due to its mucoadhesive properties.
- To achieve this, it is necessary to first explore the preparation of several hydrophilic, biodegradable homo- and copolymers by polymerization procedures compatible with the chemical nature of GG. Due to the versatility of click chemistry (CC) and in search of an orthogonal chemical procedure in IPN formation, the thiol-ene click reaction is selected to prepare and characterize various homo- and copolymers. Several batches will be synthesized to optimize polymerization conditions and, hence, choose the polymeric systems with the best performance. This is the first time that this methodology for the preparation of IPNs has been investigated.
- Synthesize a monomer that can enhance the biodegradability of the final matrices under the acidic conditions of the stomach. The compound, named acetaldehyde diallylacetal, is chosen as one of the diallyl monomers to be used in semi-IPN preparation so that the presence of the acetal group in its structure will be responsible for the increase in the biodegradability of the semi-IPN-based matrices.
- Conduct a proof of concept on the ability of the new materials to behave as AMOX controlled-release matrices at pHs 1.2 and 5.0.

## 2. Results

### 2.1. Synthesis of Acetaldehyde Diallyl Acetal (B Monomer)

**Scheme 1:**
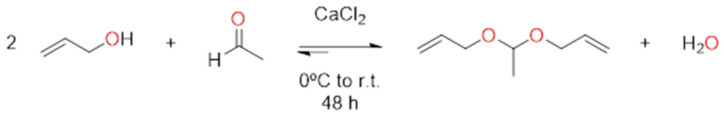
Synthetic scheme of acetaldehyde diallylacetal (B monomer).

**Figure 2:**
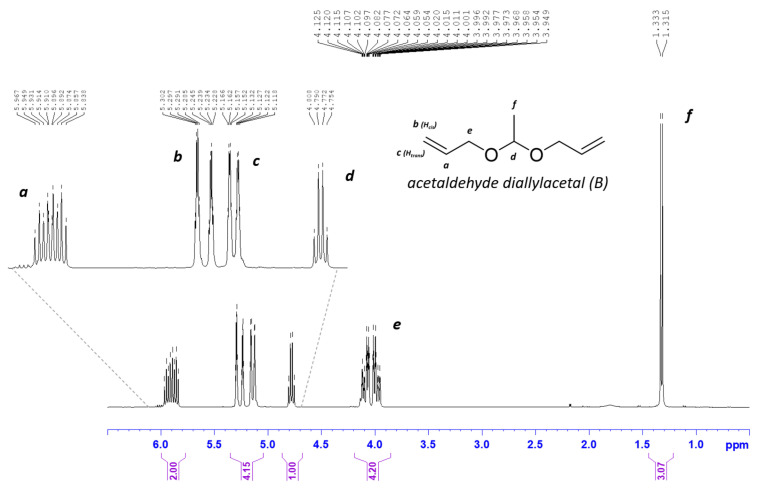
^1^H-NMR of acetaldehyde diallylacetal (B monomer) at 300 MHz in CDCl_3_.

The synthesis of the B monomer was conducted by a classic reaction of acetal formation [23] (Scheme 1), in which an equilibrium is generated, so working in the absence of moisture was essential. The title compound was isolated as a pure oil after distillation, as demonstrated by ^1^H-NMR (Figure 2) and ^13^C-NMR spectra.

The biodegradability of the B monomer (acetaldehyde diallylacetal) in the presence of acidic fluids, such as those found in the stomach, has been confirmed and will be discussed below. 

### 2.2. Preparation and Characterization of Homopolymers and Copolymers by Thiol-Ene Reaction

**Figure 3:**
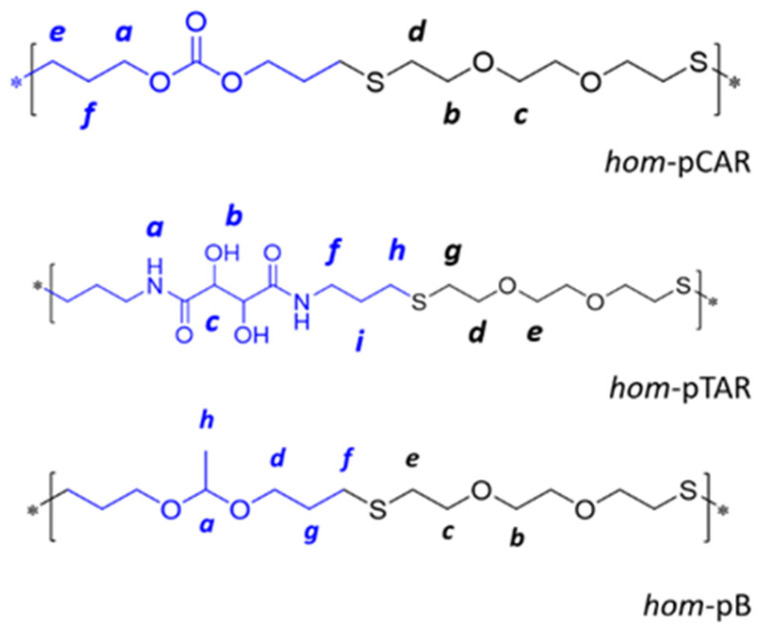
Schematic representation of constitutional repeating units for *hom*-pCAR, *hom*-pTAR, and *hom*-pB. In the case of the copolymers, the repeating units were those found in *hom*-pCAR and *hom*-pB in varied ratios, depending on the feed.

For the preparation of 400 mg of each polymer (repeating units of such polymers are recorded in Figure 3), the appropriate amount of dithiol and diallyl monomers was mixed so that the mmoles of the diallyl monomers were identical to those of 2,2′-(ethylenedioxy)diethanethiol (EDDET) in the feed for every polymerization procedure (Table 1). Homopolymers (*hom*) and copolymers (*co*) were synthesized using diallyl carbonate (CAR, commercial), (*2R,3R*)-*N,N′*-diallyltartramide (TAR, commercial), and/or acetaldehyde diallylacetal (B, synthesized de novo) as diallyl monomers, and EDDET (commercial) as the dithiol monomer. Polymerization took place in the absence of oxygen and, by means of the thiol-ene click reactions, initiated photolytically (photoinitiator: 2,2-dimethoxy-2-phenylacetophenone, DMPA). For homopolymer preparation, only one diallyl monomer was incorporated in the feed, leading to only one type of repeating unit in the polymer (Figure 2), whereas in the case of the copolymers, two diallyl monomers were used and, hence, the copolymers were constituted by two different repeating units (Figure 2). The reagents and solvents used for each case and the polymerization conditions are summarized in Table 1. 

The resulting homopolymers and copolymers were analyzed by nuclear magnetic resonance (NMR), thermogravimetric analysis (TGA), and gel permeation chromatography (GPC). The NMR spectra were used to confirm the composition of the polymers and calculate the ratio of the constitutional repeating units (CRUs) in the copolymers (Figure 2). GPC and TGA data from the copolymers are summarized in Table 2.

The NMR spectra of the synthesized homopolymers confirmed that they possess the expected chemical structure according to their repeating unit. The ^1^H and ^13^C-NMR peaks for each homopolymer are recorded below: 

*Hom*-pCAR ^1^H-NMR (300 MHz, CDCl_3_) δ ppm: 4.23 (t, 4H, H-a, *J*_a,f_ = 6.4 Hz), 3.65 (t, 4H, H-b, *J*_b,d_ = 6.9 Hz), 3.62 (s, 4H, H-c), 2.72 (t, 4H, H-d), 2.65 (t, 4H, H-e, *J*_e,f_ = 7.2 Hz), 2.02–1.89 (m, 4H, H-f). ^13^C-NMR (75 MHz, CDCl_3_, *hom*-pCAR) δ ppm: 155.1 (C=O), 71.9, 71.0 (C-b), 70.6, 70.3 (C-c), 66.5, 66.3 (C-a), 31.7, 31.5 (C-d), 29.4, 28.8, 28.7 (C-e, C-f).

*Hom*-pTAR ^1^H-NMR (300 MHz, (CD_3_)_2_SO) δ ppm: 7.73 (t, 2H, H-a, *J*_a,f_ = 5.7 Hz), 5.47 (d, 2H, H-b, *J*_b,c_ = 7.2 Hz), 4.22 (d, 2H, H-c), 3.55 (t, 4H, H-d, *J*_d,g_ = 7.0 Hz), 3.53 (s, 4H, H-e), 3.28–3.05 (m, 4H, H-f), 2.64 (t, 4H, H-g), 2.57–2.40 (m, 4H, H-h), 1.68 (q, 4H, H-i, *J*_f,i_ = *J*_h,i_ = 7.2 Hz). ^13^C-NMR (75 MHz, (CD_3_)_2_SO, *hom*-pTAR) δ ppm: 172.4 (C=O), 73.0 (C-c), 70.6 (C-d), 70.0 (C-e), 38.0 (C-f), 31.1 (C-g), 29.9 (C-i), 29.4 (C-h).

*Hom*-pB ^1^H-NMR (300 MHz, CDCl_3_) δ ppm: 4.77–4.58 (m, 1H, H-a), 3.80–3.42 (m, 12H, H-b, H-c, H-d), 2.80–2.57 (m, 8H, H-e, H-f), 1.85 (q, 4H, H-g, *J*_d,g_ = *J*_f,g_ = 6.3 Hz), 1.34–1.24 (m, 3H, H-h). ^13^C-NMR (75 MHz, CDCl_3_, *hom*-pB) δ ppm: 99.9, 99.4 (C-a), 71.6, 71.2, 71.0, 70.8, 70.3, 63.8, 63.3 (C-c, C-d), 61.5 (C-b), 32.2, 32.1, 30.1, 30.0 (C-g), 31.5, 31.4 (C-e), 29.3, 29.2 (C-f), 19.8, 19.5 (C-h).

Regarding the CAR/B copolymers, they also displayed the peaks corresponding to their expected structure. Their quantitative compositions (ratios of CRUs) were determined by ^1^H-NMR spectra analyses, comparing the integrals of peaks corresponding to protons *CH_2_-OCO-* from the CAR moiety (4.25 ppm, 4 protons per CRU) with those of the methyl protons from the B monomer (1.28 ppm, 3 protons per CRU). These data are recorded in Table 3. 

### 2.3. Degradability Studies of the B Monomer and co-p(CAR_50_B_50_) under Acidic Aqueous Conditions

**Figure 4:**
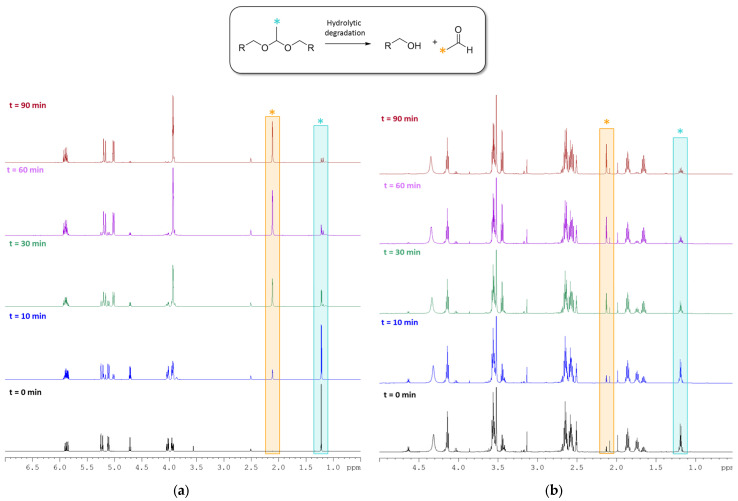
^1^H-NMR studies of the hydrolytic degradation of (**a**) B monomer and (**b**) *co*-p(CAR_50_B_50_) with time.

**Figure 5:**
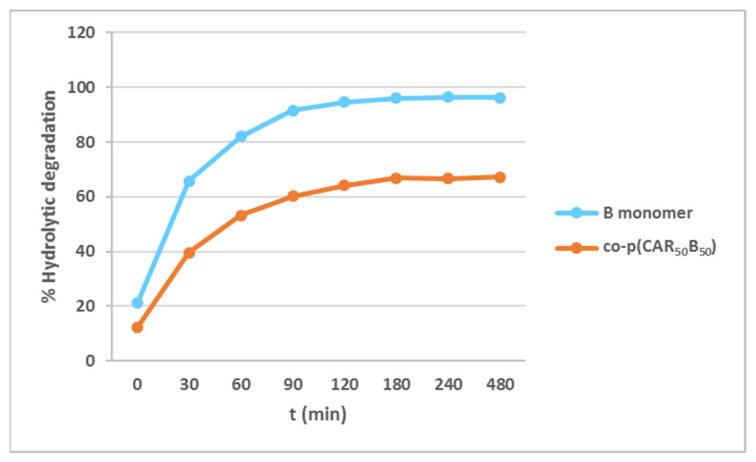
Evolution of hydrolytic degradation of the B monomer and *co*-p(CAR_50_B_50_) over time.

Hydrolytic degradation studies were followed by ^1^H-NMR spectroscopy. To simulate the acidic aqueous environment of the stomach, the samples were dissolved in a mixture of DMSO-*d_6_*/D_2_O/TFA- and the experiments conducted at 37 °C. The integral of the peak corresponding to the methyl group linked to the acetal moiety (either in B, or in *co*-p(CAR_50_B_50_)) was compared with that from the methyl group of the degradation product, i.e., acetaldehyde. Figure 4 shows the ^1^H-NMR spectra of both the degradation studies of the B monomer (Figure 4a) and of the copolymer *co*-p(CAR_50_B_50_) (Figure 4b) at fixed times: 0, 10, 30, 60, and 90 min. The results obtained in the hydrolytic degradation trials are shown in Figure 5.

### 2.4. Preparation of Guar-Gum-Based Semi-IPNs

Once the versatility of the polymerization process was optimized and the degradability properties of the new materials confirmed, five types of semi-IPNs containing GG (Polymer 1) and the thiol-ene polymer (Polymer 2) were prepared. The cross-linked Polymer 2 was grown in a colloidal aqueous suspension of Polymer 1 so that the final materials possessed Polymer 1 and Polymer 2 molecular chains that were tightly intertwined. The optimal concentration of GG in this kind of network has been established to be ca. 4% [13]. The degree of cross-linking for Polymer 2 was fixed to 8% based on the percentage of allyl groups joined to the cross-linker. Tritiol trimethylolpropane tris(3-mercaptopropionate) (TriT) was used as a covalent cross-linker. The amounts of EDDET, CAR, TAR, and/or B added to form each semi-IPN, as well as that of the covalent cross-linker TriT (dissolved in MeOH; concentration: 368.5 mg/mL), are recorded in Table 4. All the samples displayed a Polymer 1/Polymer 2 ratio of 1:1 in weight, and they were bench-stable for weeks.

### 2.5. Characterization of Guar-Gum-Based Semi-IPNs

The rheological properties of the selected semi-IPNs (GG-p(TAR_50_B_50_), GG-p(CAR_50_B_50_), and GG-pB) were evaluated. The rheological characterization encompassed strain and frequency sweep tests. Firstly, strain sweep tests (0.002–0.1% at 1 Hz) were carried out to determine the linear viscoelastic range and critical strain of the samples. Then, frequency sweep tests (0.02–10 Hz, at a constant strain within the linear viscoelastic range) were performed to analyze the stability of the samples. In these tests, the values of the viscoelastic moduli are collected (Table 5, G′ and G″ corresponding to the elastic and viscous moduli, respectively) as well as the relationship between them (tan (δ) = G″/G′) and the specific viscosity (η*). 

The morphologies of these three semi-IPNs (GG-p(TAR_50_B_50_), GG-p(CAR_50_B_50_), and GG-pB) were studied by scanning electron microscopy (SEM). Before SEM observations, the samples underwent critical point drying to prevent the alteration of their surface topography [24].

The porosity of the systems and their free volume were evaluated from the resulting SEM images. In this way, a digital processing free software, FIJI Image-J (National Institutes of Health, Bethesda, MD, USA), was used to determine the mean pore size of the selected hydrogels. Pore diameters were calculated manually by measuring 50 pores in each sample, and they were consistently identical for all of them (diameters ca. 0.290 μm).

### 2.6. AMOX Loading and In Vitro Drug Release Studies

A preliminary dissolution study using a *co*-p(CAR_50_B_50_)-based semi-IPN was performed as a proof of concept. AMOX was loaded into the matrix (10% of the total weight of the formulation).

The release studies were conducted in simulated gastric fluid sine pepsin at pH 1.2 (SGF-1.2) and pH 5.0 (SGF-5) at 37 °C. 

Drug release was determined by UV spectroscopy at 229 nm using an ex-profeso-prepared calibration curve. The release of AMOX was measured over 7.5 h, finding that there was no burst release in the first minutes, which supports the adequacy of the encapsulation of the antibiotic; on the other hand, the release of AMOX was dependent on the pH of the medium. 

## 3. Discussion

The target of the current work is the development of alternative polymeric materials that possess optimal tailor-made properties to be used as matrices in GRDDS-based formulations. Thus, since high mechanical strength is required to keep them intact throughout their stay in the stomach [25], the preparation of tightly intertwined semi-IPNs was addressed, in which GG was one of the polymers of the dual systems. The tightly intertwined structure at the molecular level of the semi-IPNs will also ensure the porosity of the final materials and, therefore, their capability to remain buoyant in gastric fluids [6], a key feature for matrices used in GRDDSs since floating systems are less likely to be expelled from the stomach compared to others [26]. Moreover, such porosity will guarantee the large encapsulation capacity of APIs (a necessary property when the controlled release of drugs at high doses is required, as is the case of AMOX) and high swelling capacities [27], which ensure the flow of gastric fluids within them. It has also been demonstrated that drug release is dependent on the swellable/erodible properties of the matrices [12]. The permanence in the stomach of these semi-IPN-based formulations can be boosted by enhancing their bioadhesive properties [21]. GG, a natural biodegradable polysaccharide, has been demonstrated to display high bioadhesive properties [22], which will impart this feature to the final matrices formed. 

There are two main strategies that have been tested for the formation of second- and third-generation IPNs. Relevant examples, prepared for potential use in drug delivery systems, are exemplified in Table 6. In general terms, the formation of second-generation IPNs (with poorer mechanical properties than those of the third generation) is usually conducted either by the mixture of two natural polymers [28,29] or by the mixture of a natural polymer and a hydrophilic synthetic macromolecule such as poly(vinyl alcohol) (PVA) [28,30], which cross-links reversibly. 

The third-generation IPNs present improved mechanical properties since Polymer 2 grows in the colloidal suspension of Polymer 1, which guarantees a more homogeneous intertwined structure of the final material [35]. In this group of IPNs, a (semi)natural polysaccharide is typically chosen as Polymer 1, such as chitosan [31], gelatin [32], or sodium alginate [34], although hydrophilic synthetic polymers can be selected [36]. Cross-linked Polymer 2 is generally formed from (meth)acryl(ate/amide) monomers, leading to 3D, although biocompatible, non-biodegradable networks. 

The versatility of the final material, in terms of chemical and physicochemical, mechanical, and biodegradability properties, can be greatly increased if bioorthogonal procedures are employed in the formation of cross-linked Polymer 2. Thus, to generate GG-based semi-IPN matrices, Polymer 2 should be prepared by a robust synthetic tool that can be conducted in aqueous media and in the presence of biomacromolecules such as GG. Thiol-ene click reactions [37,38,39], which have been widely used for the functionalization of diverse polymeric materials, meet these requirements and are the polymerization method of choice in this work. 

To enhance the biodegradability of the designed interpenetrated matrices, the incorporation of the B monomer (acetaldehyde diallylacetal) in Polymer 2 was planned. The B monomer possesses an acetal moiety in its structure that can be hydrolyzed at low pH and, therefore, can degrade in the presence of the acidic fluids found in the stomach. Thus, the ex profeso preparation of acetaldehyde diallyalacetal was first carried out. 

### 3.1. Preparation and Characterization of Homopolymers and Copolymers

**Scheme 2:**
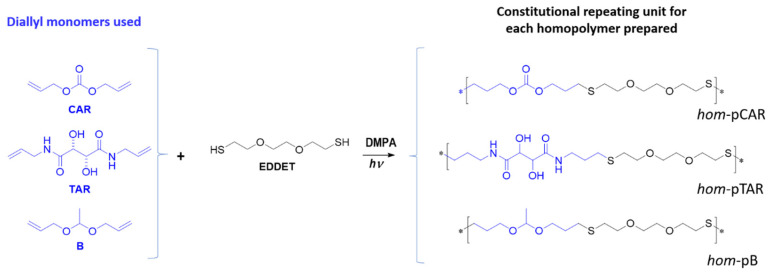
General preparation scheme of homopolymers from CAR, TAR, B, and EDDET.

The proposed thiol-ene click reaction for its future use in the formation of hydrogels (Scheme 2) was initially tested in the chosen monomers for this study, i.e., B, CAR, and TAR as diallyl monomers, and EDDET as the dithiol monomer. The reaction proceeded by means of a step polymerization procedure photoinitiated by DMPA (UV light, λ = 365 nm, P = 180 W, 10 min, then 48 h without UV light at 18 °C) to form linear polymers, as displayed in Scheme 2. The polymerization reactions were conducted with a stoichiometric ratio of allyl and thiol groups from the monomers. This type of click reaction has been demonstrated to be very versatile in the functionalization of new materials with biochemical applications [38,39]. The use of combinations of water with the hydrophilic MeO PEG-OH (*M_n_* 350) as a reaction medium for the preparation of IPN guarantees the solubility of the reagents and eliminates the use of organic solvents, which is highly relevant in achieving a procedure with an almost zero ecological impact.

The chemical compositions of the resulting homopolymers (*hom*-pCAR, *hom*-pTAR, and *hom*-pB) were confirmed by ^1^H-NMR and ^13^C-NMR spectra, and the assignments for each proton and carbon from the repeating units are detailed in the Results section.

The average molecular weights of homopolymers were determined by GPC, and the data are recorded in Table 2. The average molecular weights (*M_w_*) ranged from 42,600 for *hom*-pCAR to 19,800 for *hom*-pB. From the GPC data, it can be inferred that the degree of polymerization for *hom*-pCAR was significantly greater than those found for *hom*-pTAR and *hom*-pB. This demonstrates that, under the polymerization conditions tested, CAR was the diallyl monomer that performed best.

The thermal stability of the homopolymers was determined by TGA studies, which revealed that all of them were thermally stable when working at temperatures below 200 °C. Out of the three tested homopolymers, *hom*-pTAR exhibited the highest onset decomposition temperature $(T_{d}^{0}$= 277.9 °C, Table 2). An almost complete weight loss (>98.5%) was found in the thermal degradation profiles of polymers *hom*-pCAR and *hom*-pB when heated up to 600 °C (heating ramp: 10 °C/min) in contrast to *hom*-pTAR (% remaining weight = 11.3%). 

In summary, GPC, TGA, and NMR studies have demonstrated that the three diallyl monomers, B, CAR, and TAR, are suitable for further use in semi-IPN formation, with preferential use for the CAR monomer over the TAR monomer. The use of the B monomer is required for imparting improved degradation properties.

Given that CAR performed better regarding the final molecular weight of the polymeric materials, the preparation of the three copolymers [*co*-p(CAR_80_B_20_), *co*-p(CAR_50_B_50_), and *co*-p(CAR_20_B_80_)] with various percentages of B in the feed (Table 1) was addressed next. It was observed that the higher the percentage of B in the diallyl monomer mixtures, the lower the molecular weight of the final copolymer, giving rise to an inferior *M_w_* than that reached for *hom*-pB when the ratios of B in the copolymer mixtures were ≥50% (Table 2).

**Figure 6:**
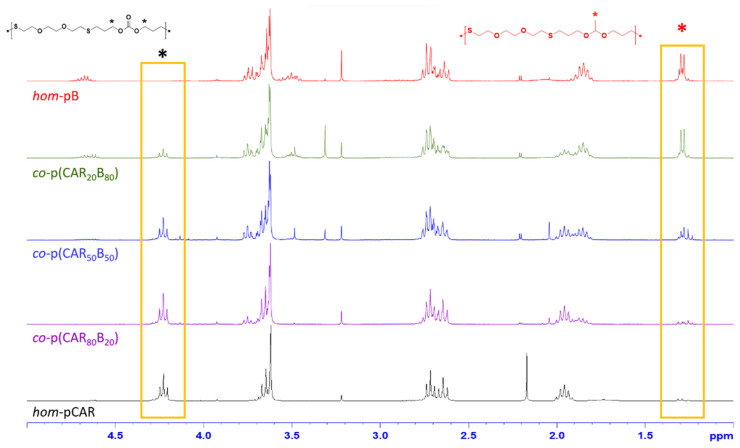
^1^H-NMR spectra of *hom*-pB, *hom*-pCAR, and CAR/B copolymers.

^1^H-NMR spectra of *hom*-pCAR and *hom*-pB, as well as the three copolymers prepared (*co*-p(CAR_80_B_20_), *co*-p(CAR_50_B_50_), and *co*-p(CAR_20_B_80_)), are shown in Figure 6. The differences in the relative intensities derived from the diallyl monomers in the copolymer composition can be easily observed. The CRUs ratios obtained by ^1^H-NMR were the result of comparing the integrals of the peaks assigned to protons CH_2_-OCO- from the CAR moiety (4.25 ppm, 4 protons per repeating unit) with those of the methyl protons from the B monomer (1.28 ppm, 3 protons per repeating unit). These data are recorded in Table 3: the calculated monomer ratios fluctuate within ±2.5–5.0% of those of the feed, underlining the excellent control over the final copolymer composition. 

### 3.2. Hydrolytic Degradation of Diallyl Moieties in B Monomer and Copolymer co-p(CAR_50_B_50_)

The hydrolytic degradation of acetal groups in the B monomer and in copolymer *co*-p(CAR_50_B_50_) was studied next. Previous studies on ionic and covalent cross-linked chitosan hydrogels for drug delivery have demonstrated that the acetal moieties of the covalent cross-linker can be bio-hydrolyzed with the concurrence of enzymatic catalysts [40]. In the present work, the degradation studies were conducted under slightly acidic conditions (pH *ca.* 5.0) and without the use of enzymatic catalysts. The experiments were carried out at 37 °C for 8 h and followed by ^1^H-NMR spectroscopy (t: 0, 0.5, 1.0, 1.5, 2.0, 3.0, 4.0, and 8.0 h). NMR tubes were loaded with the sample dissolved in a mixture of deuterated solvents: dimethylsulfoxide (DMSO-*d_6_*) and D_2_O, with a drop of trifluoroacetic acid (TFA-*d*). The results are recorded in Figure 3 and Figure 4. 

First, hydrolytic assays were conducted in aqueous media at 37 °C without the addition of acid. Both the monomer and the copolymer were stable for days under those conditions, keeping their chemical integrity. Of note was the rapid cleavage of the acetal moiety in the B monomer, which evolved to almost complete degradation within 2 hours. In contrast, when the B monomer was incorporated into a polymeric structure, even at ratios as high as 50%, the degradation rate decreased markedly. These findings were not surprising because steric hindrance caused by polymer chains was certainly expected to interfere with the degradation process. Moreover, this effect is probably more pronounced when the synthetic polymer (Polymer 2) is part of the semi-IPN system. On the other hand, these results do not have to detract from the performance of the polymeric matrices as GRDDSs; it can be inferred from the fact that the biodegradation rate is reduced that the integrity of these semi-IPN-based drug formulations is maintained for longer periods, and, therefore, drug release can be prolonged. This is in line with one of the objectives in GRDDS preparation. 

Furthermore, additional degradation assays of the polymer have been conducted in the presence of AMOX and have been followed by NMR. Two facts have emerged from such experiments: first, the polymer remains biodegradable under acidic conditions; second, the β-lactam ring (the antibiotic pharmacophore) maintains its integrity throughout the assay period (8 hours), and its -CH- groups are visible in the ^1^H NMR spectra at δ 5.51 and 4.60 ppm.

It is anticipated that changing the percentages of the B monomer in the feed when preparing semi-IPNs will provide matrices with tailor-made degradation patterns that will lead to varied drug release profiles. 

### 3.3. Synthesis and Characterization of Guar-Gum-Based Semi-IPNs

The biopolymer used for the preparation of the semi-IPN hydrogels was GG in all the trials due to its mucoadhesive properties mentioned above. The synthetic polymer chains will grow and cross-link within the GG colloidal solution to improve its final mechanical and structural characteristics. The interpenetration of this second polymer within the GG colloidal suspension is essential to provide GG-based preparations with the required consistency to host the drug and work properly as a sustained-release GRDDS without falling apart over several hours. In this way, the generation of a semi-IPN by the inclusion of a cross-linked second polymer results in a more stable system without chemically modifying GG, so its mucoadhesive properties stay untouched.

**Figure 7:**
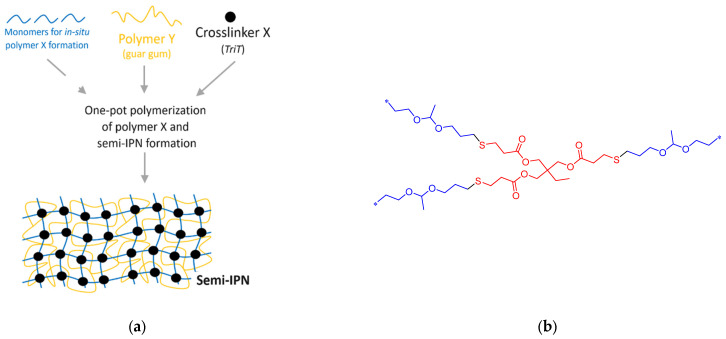
(**a**) Schematic representation of semi-IPN formation. (**b**) Example of the cross-linking point in the 3D framework by reaction of TriT with the diallyl B monomer.

Three semi-IPN matrices (GG-p(TAR_50_B_50_), GG-p(CAR_50_B_50_), and GG-pB) were designed in order to study and compare their rheological properties and microporous structure. Its general structure is shown in Figure 7a. Two key differences must be highlighted between the synthesis of these systems and that of homo- and copolymers: (1) GG was added to the feed as a natural, biodegradable polymer, thus allowing its interpenetration with in-situ growing Polymer 2; and (2) TriT acted as a covalent cross-linker to generate the synthetic network of Polymer 2. An example of a cross-linking point involving TriT and the B monomer is shown in Figure 7b. All of it makes the final structure of the product resemble a 3D network that is expected to be capable of accommodating the target drug.

**Figure 8:**
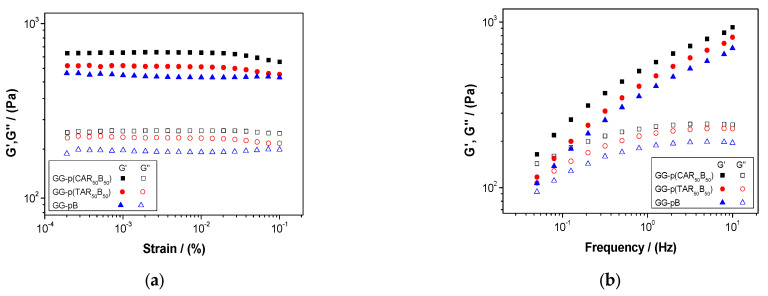

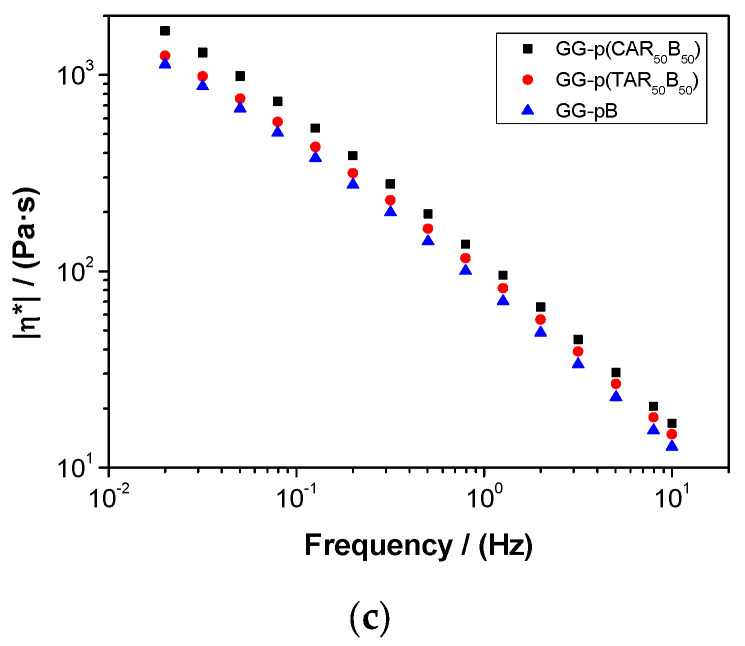
(**a**) Strain sweep tests of the different systems evaluated: GG-p(CAR_50_B_50_), GG-p(TAR_50_B_50_), and GG-pB. (**b**) Frequency sweep tests of the different systems evaluated: GG-p(CAR_50_B_50_), GG-p(TAR_50_B_50_), and GG-pB. (**c**) Evolution of the viscosity over the frequency range studied for the different systems evaluated: GG-p(CAR_50_B_50_), GG-p(TAR_50_B_50_), and GG-pB.

The study of their rheological properties is of interest due to the close correlation found between such properties in hydrogels and their drug release performance when used as DDS matrices [41]. The rheological analyses started with strain sweep tests, which are plotted in Figure 8a. The GG-pB system remained constant within the strain range studied, while both GG-p(CAR_50_B_50_) and GG-p(TAR_50_B_50_) lost their linearity from 0.02% onwards. Specifically, the critical strain for each sample was calculated, and the parameters are displayed in Table 5. The semi-IPN GG-p(CAR_50_B_50_) and GG-p(TAR_50_B_50_) exhibited a lower critical strain, as evidenced before, with the loss of the linearity of their profiles.

Then, frequency sweep tests were carried out, as shown in Figure 8b. All the systems exhibited similar behavior, with high-frequency dependency due to the high slope along the frequency range. It was observed that at high frequency, G′ values were one order of magnitude greater than G″, and every sample increased its solid character when intensifying the frequency applied.

To exemplify this frequency dependency, the G′ and tan (δ) figures at three different frequency values (0.02, 1.00, and 10.0 Hz) were calculated, and the results are shown in Table 5. As can be seen, the GG-p(CAR_50_B_50_) system exhibits a higher solid character compared to GG-p(TAR_50_B_50_) and GG-pB, increasing the differences with the frequency applied (G′ values of 183, 133, and 120 Pa at 0.02 Hz and 1012, 886 and 765 Pa at 10.0 Hz for GG-p(CAR_50_B_50_), GG-p(TAR_50_B_50_), and GG-pB, respectively). This structuring of the systems with frequency is better shown with the evolution of tan (δ) values, being close to 1 at low frequency but evolving to ca. 0.2 at high frequency. Consequently, for every system analyzed, stronger gels were obtained when the stimuli were applied more frequently, as tan (δ) values became lower at higher frequencies. 

Furthermore, the evolution of the viscosity of the systems with frequency was also plotted (Figure 8c). All the semi-IPNs exhibited similar behavior, with a linear decrease with frequency. Additionally, there are no significant differences between the η*_1_ (viscosity values at 1.00 Hz) of the formulations, obtaining values around 100 Pa·s.

Even when rheological properties did not vary significantly among the evaluated systems, major differences arose when compared with the “blank” system (colloidal suspension of GG (4% *w/v*) in distilled water-PEG solutions (9:1 *w/w*)): the GG colloidal suspension did not display a solid-like performance and evolved to a biphasic solid-liquid system in less than 24 h. On the contrary, the structures of the evaluated semi-IPNs were demonstrated to be bench-stable for more than 2 months without changing their rheological properties. 

Of note is the evolution of G′ and G″ with frequency (Figure 8b) since the evaluated semi-IPNs seem to become more rigid (↑G′, = G″) the bigger the frequency of the stimuli. This may mean that the application of high-frequency stimuli generates a microstructural modification in all the systems, such as the promotion of chain entanglements, which enhances their stability and makes them stronger gels. This behavior is what can be expected from the prepared semi-IPNs, which evolve into solid-like structures, especially when strong stimuli are applied. 

The rheological properties described above were assayed at 25 °C (the commonly chosen temperature for such experiments). However, the release of AMOX from the formulations would take place at 37 °C. Hence, it was necessary to test if the rheological properties of the matrices depended on the temperature of the designed systems and or the presence of the drug in them. If the polymers involved were thermo-responsive, significant differences in such properties would be expected. There are no precedents of such behavior for GG.

**Figure 9:**
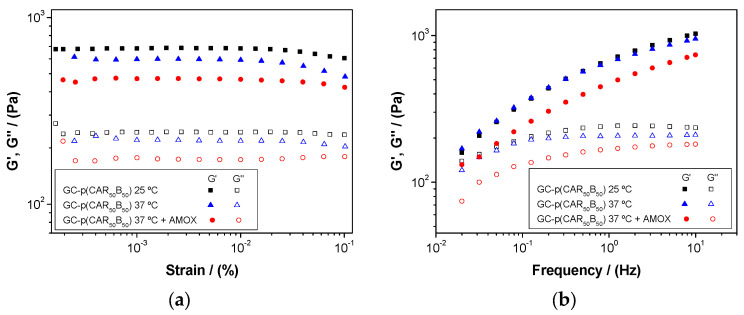
(**a**) Strain sweep tests of the different systems evaluated: GG-p(CAR_50_B_50_), at 25 and 37 °C, and the formulation of AMOX + GG-p(CAR_50_B_50_). (**b**) Frequency sweep tests of the different systems evaluated: GG-p(CAR_50_B_50_), at 25 °C and 37 °C, and the formulation of AMOX + GG-p(CAR_50_B_50_).

When submitting the same hydrogel to both strain and frequency tests at 25 and 37 °C, similar profiles were observed, showing no significant differences between them (Figure 9a,b). This proved the good resistance of the system to body temperature and, therefore, its possible application for controlled drug release treatments, as the rheological properties were very similar at 25 and 37 °C. It is worth mentioning that even though the incorporation of AMOX reduced G′ and G″ values, possibly due to a slight modification of the microstructure, the hydrogel maintained its solid-like behavior, which is fundamental for the application. This fact could be proved by comparing the values of tan (δ) obtained at 1 Hz for each system, which were very similar (0.35, 0.31, and 0.36 for GG-p(CAR_50_B5_0)_, at 25 and 37 °C and the formulation of AMOX + GG-p(CAR_50_B_50_), respectively) and showed no significant differences.

**Figure 10:**
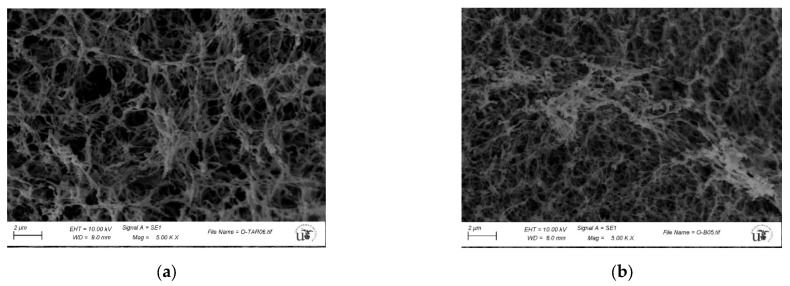

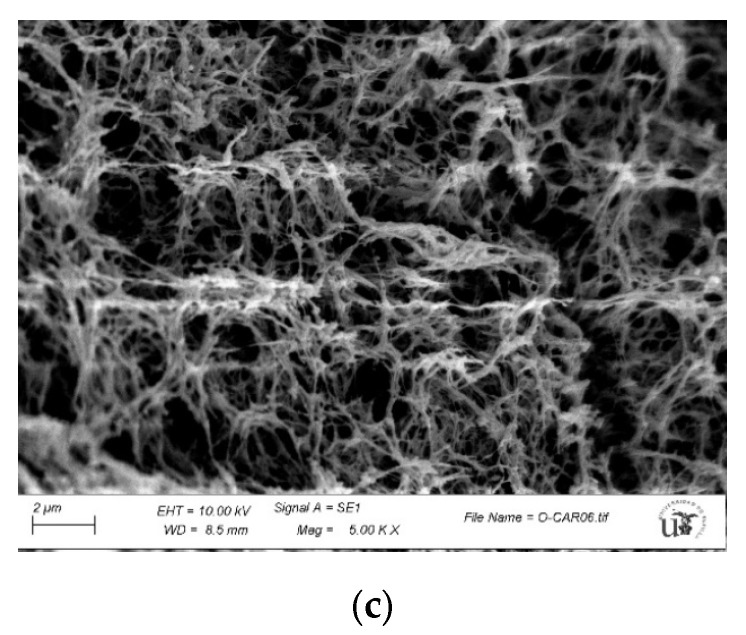
(**a**) SEM photo from GG-p(TAR_50_B_50_). (**b**) SEM photo from GG-pB. (**c**) SEM photo from GG-p(CAR_50_B_50_).

SEM observations of the networks formed confirmed the super-porous structure of the three systems, as can be observed in Figure 10. Analysis with FIJI Image-J software revealed that every sample had a similar percentage of porosity (between 22% and 29%), not finding significant differences. Although pore size was heterogeneous within every sample, their mean pore diameters were ca. 0.290 μm for all the samples (0.290 μm for [GG-p(TAR_50_B_50_)], 0.294 μm for [GG-p(CAR_50_B_50_)], and 0.288 μm [GG-pB]).

### 3.4. AMOX Loading and In Vitro Drug Release Studies

**Figure 11:**
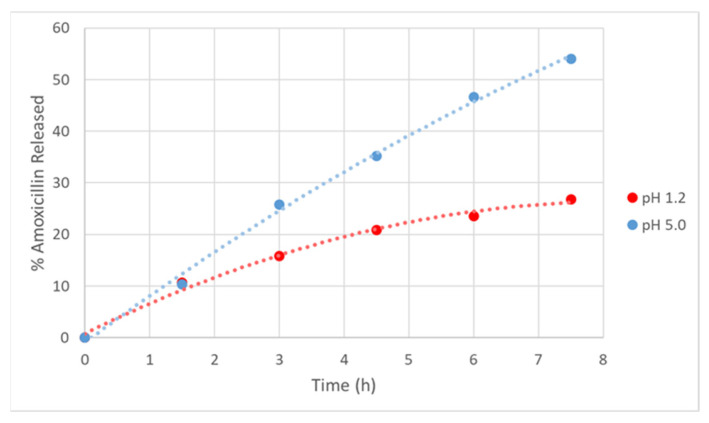
Cumulative drug release for GG-p(CAR_50_B_50_), 10% of loaded amoxicillin, at two different pH levels.

The release studies were conducted in simulated gastric fluid sine pepsin at pH 1.2 (SGF-1.2) and pH 5.0 (SGF-5) at 37 °C. The drug release was determined by UV spectroscopy (Figure 11). These assays demonstrated that (a) the synthesized systems could encapsulate the desired drug effectively since burst release was prevented, and (b) AMOX could be released from it in a controlled, sustained manner, its delivery being more rapid at pH 5.0 than at pH 1.2. In fact, cumulative drug release data approached 55% at pH 5.0 after 7.5 h, while it barely reached 30% at pH 1.2. 

Previous scientific studies regarding the release of AMOX from gastroretentive systems were generally performed at pH 1.2 as this corresponds to the normal acidity found in a healthy, empty stomach; hence, the assays were conducted at that pH for comparative reasons. Nonetheless, it cannot be forgotten that therapeutic approaches for the treatment of *H. pylori* infections tend to include an acidic-secretion-blocking compound, such as regular proton pump inhibitors or the more novel drug vonoprazan. Therefore, the expected gastric pH would inevitably be less acidic, which would justify the study of AMOX release at pH 5.0 and the consideration of these data as optimal ones in the treatment of *H. pylori* infections. Additional studies that have focused on the analyses of other parameters related to drug release from the generated semi-IPNs will be published.

## 4. Materials and Methods

### 4.1. Chemicals

Chemicals used in this work were acquired from different suppliers and used as received. Guar gum (product reference: G4129-250G), diallyl carbonate (CAR), 2,2′-(ethylenedioxy)diethanethiol (EDDET), trimethylolpropane tris(3-mercaptopropionate) (TriT), and methoxypolyethyleneglycol-350 (MeO-PEG-350) were acquired from Sigma-Aldrich (Madrid, Spain). Calcium chloride (CaCl_2_) was provided by Fisher Scientific (part of Thermo Fisher Scientific; Waltham, MA, USA). Alfa Aesar (part of Thermo Fisher Scientific; MA, USA) was the supplier of (*2R,3R*)-*N,N′*-diallyltartramide (TAR) and allyl alcohol. 2,2-Dimethoxy-2-phenylacetophenone (DMPA) and acetaldehyde were purchased from Acros Organics (part of Thermo Fisher Scientific; MA, USA). 

### 4.2. Preparation and Characterization of Homopolymers and Copolymers

B monomer (acetaldehyde diallylacetal) was prepared following the recipe described by Verdugo-Fernández [23]. The other monomers were dissolved in polymerization solvent, either ethyl acetate (EtOAc, 2 mL) or methanol (MeOH, 2 mL), depending on their solubilities. Next, the initiator (dissolved in MeOH, concentration: 340 mg/mL) was added to the monomer mixtures, and the final solutions were immediately exposed to UV light (365 nm, 180 W) for 10 min while stirred. After that, the UV light was turned off, and the reactions proceeded for 48 h at 18 °C. Once the reaction time was over, the solvent was removed under reduced pressure, yielding a transparent uncolored syrup in every case except for *hom*-pTAR, for which the new polymeric material was a white solid. The yields of the polymeric syntheses were quantitative in all cases. Additionally, NMR spectra demonstrated that there were no unreacted monomers in the polymerization media (Figure 6).

Experiments of nuclear magnetic resonance (NMR), electrospray ionization mass spectrometry (ESI-MS), and scanning electron microscopy (SEM), as well as lyophilization procedures, were conducted at the CITIUS Service (University of Seville). ^1^H-NMR and ^13^C-NMR spectra were recorded at 300 K with a Bruker—AV NEO 300 MHz for solutions in CDCl_3_ or DMSO-*d_6_*. Chemical shifts (δ) are reported as parts per million downfield from Me_4_Si and *J* in Hz. *J* is assigned and not repeated. All the assignments were confirmed by COSY and HSQC experiments. The mass spectrum of the B monomer was obtained using a Kratos MS80RFA instrument.

The thermogravimetric analyzer was a TA Instruments Q-600 SDT (New Castle, DE, USA). Platinum pans containing approximately 5 mg of each sample were used. Trials were conducted under a nitrogen atmosphere (flow rate: 100 mL/min) at a heating rate of 10 °C/min (from 25 to 600 °C), and the thermal decomposition pattern was studied. Gel permeation chromatography analyses were carried out using a Waters apparatus equipped with a Waters 2414 refractive index detector and two Styragel^®^ HR columns (7.8 × 300 mm^2^) linked in series, thermally stabilized at 40 °C, using *N,N*-dimethylformamide (DMF) containing LiBr 5.8 mM as the mobile phase (flow rate: 1.0 mL/min). Molecular weights were estimated against polystyrene standards. 

### 4.3. Preparation and Characterization of Guar-Gum-Based Semi-IPNs

The systems were prepared under an inert atmosphere. To do so, 400 mg of GG was mixed with 9 mL of deoxygenated distilled water, and the content was stirred thoroughly until a homogeneous mixture of GG and water was achieved. Next, 1 g of PEG and the corresponding amount of EDDET, CAR, TAR, and/or B (Table 4) were added to each sample, as well as the appropriate volume of a solution of the covalent cross-linker TriT (in MeOH; concentration: 368.5 mg/mL) to reach a degree of covalent cross-linking of 8%. The systems were gently mixed at 18 °C, and then the initiator was charged (dissolved in MeOH; concentration: 340 mg/mL) and homogenized. The vials were placed in a roller for 10 min while simultaneously being exposed to UV light (365 nm, 180 W). Polymerizations were allowed to proceed overnight at 18 °C in an orbital stirrer at 50 rpm. The final GG concentration that was aimed in every experiment was 4%, identical to that of the polymer synthesized de novo (Polymer 2). The presence of PEG was removed by the critical point drying method [24]; consecutive water–acetone/water–ethanol baths would ensure not only the removal of the biocompatible, non-toxic PEG but also any remaining unreacted monomer left, if necessary.

All the rheological measurements were carried out in an AR2000 rheometer (TA Instruments) using plate–plate geometry (diameter 40 mm) with a rough surface to avoid the slipping of the sample. The temperature was maintained at 25 °C using a Peltier heating system.

Images were recorded by means of a field emission scanning electron microscope, FEI Teneo, at an accelerating voltage of 5 kV using secondary electrons. The samples were dried by the critical point drying method in order to prevent the alteration of their surface topography. This protocol is widely applied for the analysis of biological tissues and was followed according to a previous study [24].

### 4.4. AMOX Loading and In Vitro Drug Release Studies

A preliminary dissolution study using the *co*-p(CAR_50_B_50_) semi-IPN was performed as a proof of concept. AMOX was loaded into the matrix by dissolving it in DMSO (concentration: 500 mg mL^−1^) and then dropping it over a 50 mg bead of the lyophilized system. The concentration for AMOX loading was chosen to be 10% of the total weight of the loaded sample. Then, the drug-loaded material was placed into dialysis tubes (1 kDa cut-off) and submerged in vessels containing simulated gastric fluid sine pepsin (prepared according to [42]) either at pH 1.2 (SGF-1.2) or pH 5.0 (SGF-5), depending on the sample. All the vessels were then placed inside an orbital incubator at 37 °C and stirred gently (100 rpm) throughout the release experiments.

At each predetermined time interval, 5 mL of the medium was withdrawn from each vessel, and its absorbance was measured at 229 nm. Dilutions of the samples were conducted when necessary. An equal volume of prewarmed, freshly prepared medium was added to the dissolution vessels after each withdrawal. The amount of AMOX released by the system was calculated by means of the calibration curve.

## 5. Conclusions

In this study, the preparation of novel semi-IPNs for their future use as matrices in GRDDSs was successfully addressed using guar gum (GG, Polymer 1) and a biodegradable synthetic copolymer (Polymer 2) as the unique components of super-porous matrices. 

The biodegradable Polymer 2 was synthesized by means of a new procedure, thiol-ene click reactions, in the design of semi-IPNs as DDSs. This polymerization process was initially tested by the preparation of different linear homopolymers using labile monomer acetaldehyde diallylacetal (B monomer), diallyl carbonate (CAR), and (*2R,3R*)-*N,N′*-diallyltartramide (TAR) as the diallyl monomers; 2,2′-(thylenedioxy)diethanethiol (EDDET) was the selected dithiol monomer in every case. The resulting polymers were characterized by GPC and NMR studies, confirming the adequacy of the process. All the evaluated macromolecules were demonstrated to be thermally stable below 200 °C (TGA analysis), while their weight average molecular weight ranged from 42,600 to 19,800, showing that CAR was the diallyl monomer that polymerized more efficiently under the tested conditions. 

The procedure to generate copolymers with varied potential degradability was investigated next. Three combinations of the diallyl monomers CAR and B were chosen (molar ratios 80:20, 50:50, and 20:80). The experimental compositions of the copolymers fitted perfectly well with the theoretical monomer ratios, as confirmed by ^1^H-NMR spectra. The acidic hydrolyses of the B monomer and the copolymer *co*-p(CAR_50_B_50_) (to be used as Polymer 2 in semi-IPNs) were investigated to confirm their hydrolysis and, hence, the future degradability of the semi-IPNs to be prepared. In total, 67% of acetal moieties from polymer *co*-p(CAR_50_B_50_) were degraded under acidic conditions in 4 hours, as corroborated by NMR studies. 

Next, semi-IPNs were synthesized using the mucoadhesive polysaccharide guar gum and different combinations of CAR/B diallyl monomers. Trimethylpropane tris(3-mercaptopropionate) (TriT) was the cross-linking agent in every case (degree of cross-linking: 8%). The rheological properties of the resulting networks were evaluated. Every system showed similar values for tan (δ) (≈0.37 at 1.00 Hz) and similar behavior (enhanced solid-like features) when the frequency of stimuli increased. Moreover, SEM observations confirmed the super-porous morphology of the systems, with a mean pore diameter of 0.290 ± 0.004 μm. Based on the overall results presented, semi-IPN formulations with CAR and B as diallyl monomers are expected to perform best as polymeric matrices for the sustained release of APIs at the gastrointestinal level. 

Preliminary loading and release assays of AMOX from the designed semi-IPNs demonstrated that AMOX could be released from it in a controlled, sustained manner. These results pave the way for the preparation of GRDDSs for the treatment of *H. pylori* infections as well as other pathologies. These mechanically strong, well-structured, mucoadhesive, super-porous semi-IPN matrices may be of use in oral drug administration as they will be capable of controlling the release of selected APIs, such as AMOX, while disintegrated under the mildly acidic conditions of the stomach of patients treated. 

## Figures and Tables

**Table 1:** Preparation of homopolymers and copolymers.

Polymer	CAR (μL)	TAR (mg)	B (μL)	Diallyl Monomers Molar Ratio	EDDET (μL)	Initiator (mg)	Solvent (mL)
*hom*-pCAR	176.8	-	-	-	200.7	31.6	2 (EtOAc)
*hom*-pTAR	-	222.4	-	-	158.6	25	2 (MeOH)
*hom*-pB	-	-	202.2	-	200.7	31.6	2 (EtOAc)
*co*-p(CAR_80_B_20_)	141.5	-	40.4	80:20	211.2	31.6	2 (EtOAc)
*co*-p(CAR_50_B_50_)	88.4	-	101.1	50:50	211.2	31.6	2 (EtOAc)
*co*-p(CAR_20_B_80_)	35.36	-	161.7	20:80	211.2	31.6	2 (EtOAc)

Polymerization conditions: [Diallyl monomers] and [EDDET] in this reaction were both 0.487 M. Reaction conditions: [diallyl monomers] = 0.616 M; [EDDET] = 0.616 M; solvent = 2 mL of either EtOAc or MeOH; initiator = DMPA 10%; T = 18 °C; UV radiation = 365 nm, 180 W, 10 min; stirred for 48 h.

**Table 2:** GPC and TGA data for synthesized homopolymers and copolymers.

Polymer	Diallyl Monomer and Molar Percentage ^a^	*M_w_* ^b^	*M_n_* ^b^	*M_w_/M_n_* ^b^	TGA ^c^		
					$T_{d}^{0}$	*T_d_* (°C)	ΔW(%)
*hom*-pCAR	CAR	42,600	33,400	1.3	268.5	340.6	98.9
*hom*-pTAR	TAR	21,900	18,000	1.2	277.9	351.3	88.7
*hom*-pB	B	19,800	16,500	1.2	226.5	350.6	99.4
*co*-p(CAR_80_B_20_)	CAR-B 80:20	21,500	18,000	1.2	253.4	339.9	100.0
*co*-p(CAR_50_B_50_)	CAR-B 50:50	11,200	8800	1.3	230.9	334.0	98.3
*co*-p(CAR_20_B_80_)	CAR-B 20:80	8100	6900	1.2	236.4	342.2	98.9

^a^ Each polymer uses EDDET as the dithiol monomer. ^b^ Determined by GPC. ^c^ Onset decomposition temperature corresponding to 10% of weight loss ($T_{d}^{0}$), maximum rate decomposition temperatures (*T_d_*), and weight loss at the respective decomposition step [ΔW(%)] determined by TGA.

**Table 3:** Experimental molar ratios of constitutional repeating units for the synthesized CAR/B copolymers.

Copolymer	Ratio Diallyl Monomers in the Feed (in Molar Percent)		^1^H-NMR Integrals for the Peaks at δ (ppm) 4.25 (from CAR Moiety: 4 H) and 1.28 (from B Moiety: 3 H)		^1^H-NMR Integrals for the Peaks at δ (ppm) 4.25 (from CAR Moiety: 4H) and 1.28 (from B Moiety: 3 H) Divided by the n.º of H for each Peak		Experimental CAR/B Molar Ratios (in %)	
	CAR	B	4.25 ppm	1.28 ppm	4.25 ppm	1.28 ppm	CAR	B
*co*-p(CAR_80_B_20_)	80	20	4 ^a^	0.6494	1	0.2165	82.1	17.9
*co*-p(CAR_50_B_50_)	50	50	4 ^a^	2.7339	1	0.9113	52.4	47.6
*co*-p(CAR_20_B_80_)	20	80	4 ^a^	10.6580	1	3.5527	21.9	78.1

^a^ The integral for the peak at 4.2 ppm was fixed to 4.0.

**Table 4:** Preparation of guar-gum-based semi-IPNs.

semi-IPN	CAR (μL)	TAR (mg)	B (μL)	Diallyl Monomers Molar Ratio	EDDET (μL)	TriT (mg)	Initiator (mg)
GG-p(TAR_50_B_50_)	-	122	87.7	50:50	160.1	22.7	27.4
GG-pB	-	-	198.1	100	180.9	25.7	31
GG-p(CAR_50_B_50_)	86.6	-	99.1	50:50	180.9	25.7	31
GG-p(CAR_80_B_20_)	138.6	-	39.6	80:20	190.4	27	31
GG-p(CAR_20_B_80_)	34.35	-	158.5	20:80	190.4	27	31

Reaction conditions: initiator = DMPA 10%; methoxypolyethyleneglycol-350 (MeO-PEG 350) = 1 g; GG = 400 mg dissolved in 9 mL of distilled water; T = 18 °C; UV radiation: 365 nm, 180 W, 10 min; reaction time: 24 h.

**Table 5:** Critical strain, elastic modulus (G′), and tan (δ) values at 0.02, 1.00, and 10.0 Hz for the different systems evaluated.

Systems	Critical Strain (%)	G′ (Pa)			tan (δ)		
		0.02 Hz	1.00 Hz	10.0 Hz	0.02 Hz	1.00 Hz	10.0 Hz
GG-p(CAR_50_B_50_)	0.012 ± 0.003	183 ± 34	681 ± 51	1012 ± 22	0.81 ± 0.09	0.35 ± 0.02	0.23 ± 0.01
GG-p(TAR_50_B_50_)	0.012 ± 0.003	133 ± 24	574 ± 48	886 ± 21	0.88 ± 0.06	0.39 ± 0.03	0.25 ± 0.01
GG-pB	0.086 ± 0.020	120 ± 20	495 ± 40	765 ± 20	0.85 ± 0.05	0.38 ± 0.03	0.24 ± 0.01

**Table 6:** Selected examples of IPN matrices prepared for their use as DDS.

Entry	Type of IPN	Drug	POLYMER 1		POLYMER 2		Polymerization Method	Ref.
			(A) Polysaccharides (B) p(Meth)acryl(ate/amide)s (C) Others	(A) Xrlinked? (Y/N) (B) if Yes, Xrlinker	(A) Polysaccharides (B) p(Meth)acryl(ate/amide)s (C) Others	(A) Xrlinked? (Y/N) (B) if Yes, Xrlinker		
**1**	2nd-Generation Semi-IPN	Diclofenac sodium	(A) Sodium Alginate	Y; Ca^2+^ ions (from CaCl_2_)	(A) Guar Gum, Xanthan gum, Gellan gum (C) PVA	N; ---	--- Physical mixture and then ionic gelation method	[28]
**2**	2nd-Generation Full-IPN	Amoxicillin	(A) *Sterculia foetida*	Y; Glutaraldehyde	(A) Pullulan	Y; Glutaraldehyde	Physical mixture and then Xrlinked together	[29]
**3**	2nd-Generation Full-IPN	Esomeprazole	(A) CTS	Y; Glutaraldehyde	(C) PVA	Y; Glutaraldehyde	Physical mixture and then Xrlinked together	[30]
**4**	3rd-Generation Semi-IPN	Clarithromycin (*H. Pylori*)	(A) CTS and (C) PVP, MMT	N; ---	(B) PAAm-*co*-PAA	Y; MBA	Polymer 2: Free radical polymerization (TEMED, APS)	[31]
**5**	3rd-Generation Semi-IPN	5-Fluorouracil	(A) Gelatin and (C) PVP	N; ---	(B) PAA	Y; EGDMA	Polymer 2: Free radical polymerization (APS, NaHSO_3_)	[32]
**6**	3rd-Generation Semi-IPN	Curcumine	(B) PDMAEMA	N; ---	(B) PAAm	Y; MBA	Polymer 1: Free radical polymerization (AIBN, toluene) Polymer 2: Free radical polymerization (TEMED, APS)	[33]
**7**	3rd-Generation Full-IPN	Resveratrol	(A) Sodium Alginate and Gelatin	Y; Ester formation between COOH and OH groups from polymers (coupling agent: EDC/NHS)	(B) PHEMA	Y; PEGDMA	Polymer 2: Free radical polymerization (PPS, TEMED)	[34]
**8**	3rd-Generation Full-IPN	---	(B) PDEAAm-*co*-PIAM	Y; Xrlinked with monomer IAM	(B) PDEAAm	Y; MBA	Polymer 1: Free radical polymerization (TEMED, APS) Polymer 2: Free radical polymerization (TEMED, APS)	[35]

AIBN: azobisisobutyronitrile; APS: ammonium persulfate; CTS: chitosan; EDC: *N*-ethyl-*N′*-(3-dimethylaminopropyl)carbodiimide hydrochloride; EGDMA: ethyleneglycol dimethacrylate; *H. pylori*: *Helicobacter pylori*; IPN: interpenetrated networks; MBA: *N,N′*-methylenebisacrylamide; MMT: montmorillonite; NHS: *N*-hydroxysuccinimide; PAA: poly(acrylic acid); PAAm: poly(acrylamide); PDEAAm: poly(*N,N*-diethylacrylamide); PDMAEMA: poly(*N,N*-dimethylaminoethyl methacrylate); PEGDMA: polyethyleneglycol dimethacrylate; PHEMA: poly(2-hydroxyethyl methacrylate); PIAM: poly(itaconamic acid); PPS: potassium persulfate; PVA: poly(vinyl alcohol); PVP: polyvinylpirrolidone; TEMED: *N,N,N′,N′*-tetramethylethylenediamine; Xrlinked: cross-linked; Xrlinker: cross-linker.

## Data Availability

Not applicable.
